# A perspective on the major light-harvesting complex dynamics under the effect of pH, salts, and the photoprotective PsbS protein

**DOI:** 10.1007/s11120-022-00935-6

**Published:** 2022-07-10

**Authors:** Eleni Navakoudis, Taxiarchis Stergiannakos, Vangelis Daskalakis

**Affiliations:** Department of Chemical Engineering, Cyprus University of Technology, 95 Eirinis Street, 3603 Limassol, Cyprus

**Keywords:** LHCII, Light-harvesting complex, NPQ, Photoprotection, Molecular dynamics, Dissipating state

## Abstract

The photosynthetic apparatus is a highly modular assembly of large pigment-binding proteins. Complexes called antennae can capture the sunlight and direct it from the periphery of two Photosystems (I, II) to the core reaction centers, where it is converted into chemical energy. The apparatus must cope with the natural light fluctuations that can become detrimental to the viability of the photosynthetic organism. Here we present an atomic scale view of the photoprotective mechanism that is activated on this line of defense by several photosynthetic organisms to avoid overexcitation upon excess illumination. We provide a complete macroscopic to microscopic picture with specific details on the conformations of the major antenna of Photosystem II that could be associated with the switch from the light-harvesting to the photoprotective state. This is achieved by combining insight from both experiments and all-atom simulations from our group and the literature in a perspective article.

## Τhe macroscopic view: photoprotection

Photosynthesis is a natural process that relies on solar energy harvesting and its transformation into chemical energy in order to sustain most life and primary production on earth (Blankenship 2014; Croce and van Amerongen 2020). Due to the diurnal cycle and the environmental conditions, large fluctuations are induced in the solar light intensity and its spectral distribution (Scholes et al. 2011). How do the photosynthetic organisms survive under such an unreliable source of energy as sunlight? The answer lies in a well-orchestrated and modular network of proteins and pigments (chlorophylls and carotenoids). In higher plants, the reaction centers reside within the cores of large protein assemblies called Photosystems I (PSI) and II (PSII), where the production of chemical energy is initiated. Photons are captured by peripheral antennae, the light-harvesting complexes (LHCs), which are assemblies of pigment-binding proteins. Specifically, the PSII complex is surrounded by the CP24 (polypeptide lhcb6), CP26 (lhcb5), and CP29 (lhcb4) monomers called minor antennae, and the LHCII trimers called major antennae (polypeptides lhcb1-3) (Su et al. 2017a; Chukhutsina et al. 2020). These modular components of the photosynthetic apparatus of higher plants are densely packed within the crowded thylakoid membrane environment, where they are allowed to re-organize and interact with each other. This dynamic re-organization of the thylakoid membrane is crucial for an efficient light-harvesting process in diverse environmental conditions (Johnson et al. 2011a, b; Duffy et al. 2013; Rochaix 2014). For example, the functional antenna size of PSI/II can be efficiently regulated in this consensus to adapt to the environmental conditions as a short-term response, while at the same time chlorophylls are densely packed avoiding concentration quenching (Ruban 2016; Ruban and Saccon 2022). Thus, a key first property one must explore is the mobility of the antenna complexes within the thylakoid membrane (first objective).

As we further zoom into the components of the photosynthetic apparatus, light is absorbed by the pigments that get excited within the LHCs. Thus, excitation energy from the absorbed photons is funneled downhill from the periphery of the PSI/II to the reaction centers (Croce and van Amerongen 2020). Higher plants are able to cope with the light intensity variations and sustain their homeostasis by the fine-tuning of the absorption of light and the economic funneling of the excitation energy toward the reaction centers of the photosynthetic apparatus (Pascal et al. 2005; Belgio et al. 2014). A photoprotective mechanism has thus evolved in nature as a response to sudden excesses of illumination, mainly to avoid an oxidative stress. The mechanisms of safe energy dissipation as heat can be cumulatively expressed by the non-photochemical quenching (NPQ) of chlorophyll fluorescence, with an energy-dependent and rapidly reversible major component termed qE (Horton et al. 1996; Müller et al. 2001; Holt et al. 2004). This involves a quick adaptation of the thylakoid membrane to light intensity (seconds to minutes). The mechanism safely dissipates the excess energy as heat within the LHCII antenna and eases the excitation load on PSII reaction center (Demmig-Adams et al. 2014). Thus, another key property one must explore is the conformation of the antenna complexes within the thylakoid membrane. These complexes switch between light-harvesting and dissipating states (second objective).

Structural, electronic, and dynamical information on the interactions between pigments and the protein scaffold is necessary to decipher the dual role of LHCII. The high-resolution structures of PSII-LHCII (Su et al. 2017b), or PSI-LHCI-LHCII (Huang et al. 2021) super-complexes available in the literature are the ideal starting points to extract such information however the different timescales of the diverse processes involved necessitate the combination of elaborate computational methods. For the latter, we must consider that in the photosynthetic apparatus, at most one photon is absorbed per millisecond (ms). Conformational changes in the protein scaffold of LHCII also occur at a timescale of ms. Exciton Energy Transfer (EET) between individual pigments occurs at the timescale of less than a picosecond (ps), equilibration of the energy distribution within a single LHC occurs at the timescale of a few tens of ps, and at the nanosecond (ns) scale for the EET between different complexes (Croce and van Amerongen 2020). Ultimately, the excited state decays via numerous processes and the associated lifetime is around 2 ns within the thylakoid membrane. The latter is extended to 4 ns for isolated complexes.(Belgio et al. 2012) To probe such processes in our group, we employ a combination of classical molecular dynamics (MD) and enhanced sampling techniques, like the parallel tempering metadynamics at the well-tempered ensemble (PTmetaD-WTE) (Bussi et al. 2006). The latter techniques along with Markov State Modeling (MSM) (Husic and Pande 2018) can decipher the conformational changes in the LHCII protein scaffold that occur at the long-timescale (ms) and could be associated with the transition between light-harvesting and dissipating states. Quantum Mechanics/Molecular Mechanics based (QM/MM, or QM-MD), and empirical methods (transition charges from electrostatic potential—TrESP) (Renger and Müh 2013; Maity et al. 2021) can decipher processes, like EET at the shorter timescales (ps).

Deciphering the NPQ mechanism and especially qE can be a milestone in the field of bioenergetics. NPQ activation is associated with conformational changes within the major LHCII antenna trimer of PSII (Saccon et al. 2020b) and a substantial decrease in quantum efficiency of photosynthesis (Kromdijk et al. 2016). Thus, fundamental research on NPQ has applications in highly efficient artificial light-harvesting devices (Barber and Tran 2013; McConnell et al. 2010; Croce and van Amerongen 2014; Chabi et al. 2017) and in crop productivity, (Bita and Gerats 2013; Kromdijk et al. 2016) both being important aspects for the survival in harsh environments under an ongoing climate change.

Herein, we present a perspective with a focus on the qE component of NPQ, (Horton et al. 1996; Müller et al. 2001; Holt et al. 2004) based on experimental insight combined with computational modeling at atomic scale. The main discussion and perspective rely on the atomic scale computational works of our group with the aim to provide an insight into the regulating mechanisms of the photosynthetic light energy input in relation to the aforementioned two main objectives (antenna mobility and conformations within the thylakoid membranes). The manuscript is organized into  six main sections as follows: (1) the major molecular players in qE, (2) the proposed quenching site, and the simulation of the enhanced ΔpH in the LHCII models, (3) the LHCII aggregating model in the re-organization of the thylakoid membrane and the role of the thylakoid lipids (first objective), (4) the conformational changes (configurational space) of the LHCII antenna trimer scaffold in relation to excess energy quenching (second objective), (5) the rigidity of the LHCII protein scaffold,  and (6) a qE induction model is proposed and discussed upon considering the state of the art in the literature, with the focus on the LHCII trimer transition from the light-harvesting to the dissipating configuration.

## Zoom into the atomic scale: the major molecular players in qE

**Fig. 1:**
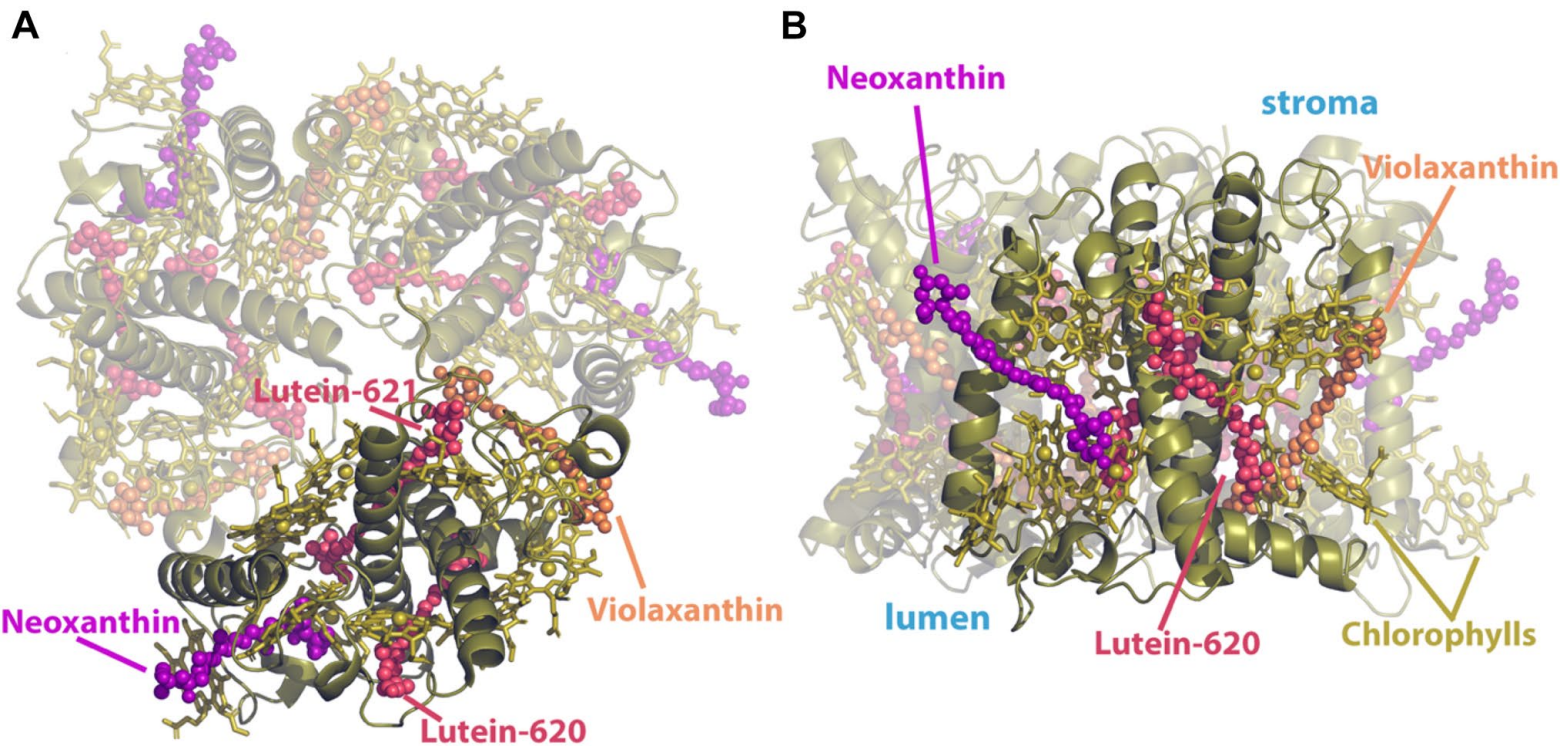
Top (**A**) and side (**B**) views of the major Light-Harvesting Complex II (LHCII) trimer. The carotenoids (neoxanthin in magenta, luteins in red, and violaxanthin in orange) are shown for reference in a ball and stick representation. Chlorophylls are shown in olive-green sticks. Only chain y (pdb ref 5XNM) (Su et al. 2017b) is shown as rigid olive-green cartoons, while the rest of the trimer chains (polypeptides) are shown as transparent moieties

Even in the absence of minor antennae, or the photoprotective protein PsbS (Photosystem II subunit S), (Croce 2015) one can have the full extent of qE, given an enhanced transthylakoid membrane proton gradient (ΔpH) (Saccon et al. 2020b). The major LHCII can thus serve as the light-harvesting, but also as the photoprotective site in photosynthesis. Each polypeptide of the LHCII trimer contains eight chlorophylls-a (chl-a), six chlorophylls-b (chl-b), two luteins (Lut-620, 621), a 9-cis neoxanthin (Neo), and a violaxanthin (Vio) within the protein scaffold as depicted in Fig. 1.

Individual LHCIIs (embedded within a thylakoid membrane) can rapidly adapt their shape (conformation) to a changing environment by switching between conformations by an inherent flexibility (Pascal et al. 2005; Schaller et al. 2011; Duffy et al. 2013; Liguori et al. 2015; Crisafi and Pandit 2017; Daskalakis et al. 2019a, 2020; Saccon et al. 2020a, b; Azadi-Chegeni et al. 2021). Even subtle conformational changes are responsible for the formation of pathways within the major LHCII that can dissipate the excess energy under increased illumination and protect the photosynthetic apparatus from oxidative damage, as an intrinsic property of LHCIIs (Ilioaia et al. 2008; Krüger et al. 2011a, b, 2012; Liguori et al. 2015; Papadatos et al. 2017; Cupellini et al. 2020; Li et al. 2020; Ruban and Wilson 2020). The transition from the light-harvesting to the quenched conformation can also be related to a subtle change in the LHCII volume of around 0.006%, (Santabarbara et al. 2009) that could be relevant to a LHCII conformational change that in turn affects the pigment network (Lapillo et al. 2020). Three main factors are reported in the literature to be correlated with qE in vivo, and consequently with the associated conformational transition in LHCII of higher plants:

(a) The acidification of the thylakoid lumen space down to a pH of around 5.5 under high illumination. The acidification comes as a consequence of the saturation of the electron transport chain (Horton et al. 1996; Holt et al. 2004). The ADP substrate of ATP-synthase is in scarcity given the high rate of light-driven photosynthetic reactions under high illumination, and the synthase is rendered incapable of pumping protons from the lumenal to the stromal space (Kanazawa and Kramer 2002). As a result, an enhanced transthylakoid membrane ΔpH is developed and protonations of lumen-exposed residues of LHCII, like aspartate or glutamate, are expected under these conditions (Ruban et al. 1996).

(b) The de-epoxidation of violaxanthin on the periphery of LHCII to zeaxanthin (Zea) via the xanthophyll cycle at low lumenal pH (Demmig-Adams and Adams 1996; Niyogi et al. 1997; Xu et al. 2015). The presence of the most hydrophobic xanthophyll (Zea) within or on the periphery of LHCII could alter the pKa values of its lumen-exposed residues and in turn induce their protonations at relatively moderate ΔpH values, relevant for the in vivo trigger of qE (Ruban et al. 2012). Zea has been found to be associated with the slow relaxation of qE, (Ruban et al. 2012) and to enhance quenching by inducing conformational changes in LHCII toward a transition into an aggregated state (Shukla et al. 2020). A change in the rigidity of the thylakoid membrane, in the presence of unbound Zea, could also increase the lateral membrane pressure on the hydrophobic membrane, alter protein-lipid interactions and induce conformational changes in LHCII to force the trimer to switch to the dissipating state (Tietz et al. 2020; Azadi-Chegeni et al. 2022).

(c) The photoprotective protein PsbS which interacts with both the minor and the major LHCII antennae (Dall’Osto et al. 2017; Sacharz et al. 2017) could also regulate the pKa values of lumen-exposed residues of the LHCII, tune the sensitivity of LHCII to protons, and affect the mobility of proteins within the thylakoid membrane. The spatial reorganization and conformational changes of the major LHCII in the presence of PsbS, within the thylakoid membranes, or changes in the membrane morphology are still a matter of debate (Duffy et al. 2013; Steen et al. 2020; Ruban and Wilson 2020). Without assuming a direct role in energy quenching, PsbS can control the amplitude of qE, (Li et al. 2002; Johnson et al. 2011b; Johnson and Ruban 2011; Goral et al. 2012) by affecting the LHCII conformation and possibly by inducing a change in the orientation of luteins within LHCII. (Son et al. 2003; Wentworth et al. 2003) PsbS has also been proposed to take the role of a ‘membrane lubricant’ that induces protein mobility, because the lack of PsbS (npq4 mutant) promotes the formation of ordered (crystalline) phases, where proteins are immobilized (Goral et al. 2012). PsbS has been shown to quickly respond to pH changes and act as an efficient sensor of light fluctuations, (Ruban et al. 2012; Ruban 2016, 2018) by easily altering its conformation in order to bind to LHCII (Daskalakis and Papadatos 2017; Liguori et al. 2019). Therefore, the PsbS effect can be more complex than a simple first order induction of the LHCII dissipating state (Steen et al. 2020).

Two factors (Zea and PsbS) are required for the in vivo normal operation of qE at physiological lumenal pH values of around 5.5 and have been associated with allosteric effects on this line (Nicol et al. 2019). In the absence of Zea, the activation of qE necessitates the drop of the pH value in the lumen to 4.7–5.0, (Ruban et al. 2012) which would induce a denaturation of the photosynthetic proteins. Zea was also found to enable an efficient binding between LHCII and PsbS in the photoprotective state (Daskalakis and Papadatos 2017; Correa-Galvis et al. 2016; Sacharz et al. 2017). Simulations indicate that LHCII conformational changes under qE conditions can be observed when the majority of protonable residues at the lumenal side of LHCII are protonated, in the absence of other external stimuli, (Ioannidis et al. 2016; Papadatos et al. 2017; Daskalakis et al. 2019a, 2020) and can be associated with qE as already reported herein at enhanced transthylakoid ΔpH, in the absence of Zea, or PsbS (Saccon et al. 2020b). Thus, we can track down the onset of qE only at the conformational changes within the major LHCII under an enhanced enough transthylakoid membrane ΔpH. In other words, ΔpH is the necessary and sufficient condition to promote these changes, whereas Zea and PsbS, with additive effects, (Johnson and Ruban 2011) can regulate the sensitivity of qE to ΔpH. This can favor the dissipating LHCII state, without excluding also the possibility that PsbS, Zea enable the LHCII aggregation, or additional conformational changes in LHCII in vivo toward the dissipating state.

All players discussed herein and associated with the onset of qE should affect, as already reported, the conformation of the major antenna complex LHCII. Changes in the conformation of the LHCII protein scaffold due to ΔpH, PsbS, and Zea should also be communicated to the pigment network. A change in the interpigment interactions in the latter network should enable the formation of energy traps, or quenching sites where the excess absorbed energy is dissipated as heat.

## Zoom into the atomic scale: the quenching site

**Fig. 2:**
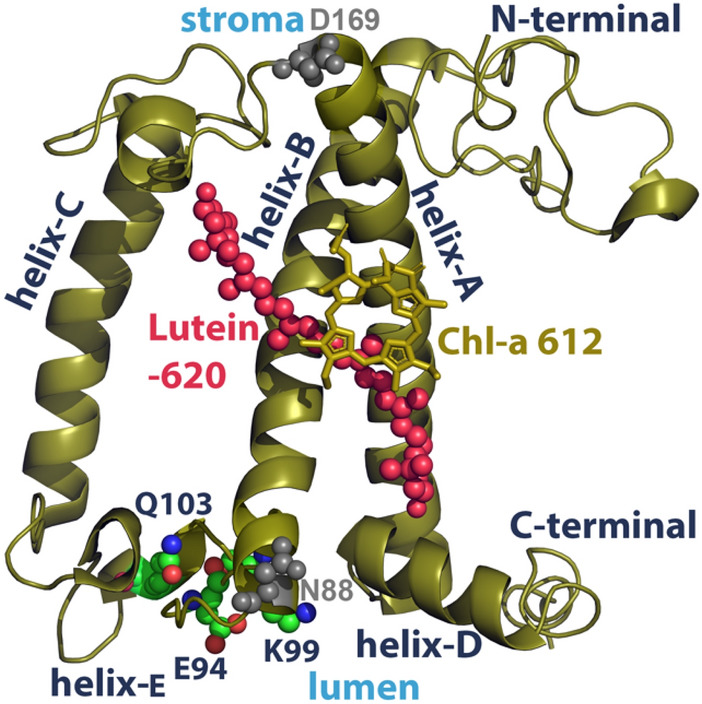
The chain y monomer (pdb ref 5xnm) of the major LHCII trimer in οlive-green cartoons. Lutein-620 and Chl-a 612 are shown for reference in red spheres and olive-green sticks, respectively. Selected polypeptide domains are indicated as helices A/B/C/D, C-terminal, and N-terminal. The triad of residues Gln-103 (Q103), Glu-94 (E94), and Lys-99 (K99) is also shown in spheres at the lumenal side (green for carbon, red for oxygen, and blue for nitrogen atoms). Residues Asn-88 (N88) and Asp-169 (D169) are shown in gray for reference

There is experimental evidence that the site of quenching (qE) lies solely within the major LHCII antenna trimer that is normally associated with PSII (Nicol et al. 2019; Saccon et al. 2020b; Ruban and Wilson 2020). Specifically, the interaction between chlorophylls and carotenoids within LHCII, that involves either energy transfer from the Chl Qy to the short-lived dark S_1_, S* carotenoid states, or charge transfer (CT) states can efficiently dissipate the excess energy as heat (Ruban et al. 2007; Ilioaia et al. 2011; Chmeliov et al. 2015, 2016; Fox et al. 2017; Park et al. 2018; Khokhlov and Belov 2019; Maity et al. 2019; Mascoli et al. 2019; Cupellini et al. 2020; Li et al. 2020; Son et al. 2020a, b). For a concise review of the different proposed mechanisms of chlorophyll excited state quenching within the photoprotective scheme, please refer to (Ruban and Saccon 2022). Concerning the associated timescales, it has been proposed that EET between Qy and S_1_ occurs within 20–50 picoseconds, and it is mediated by a weak resonance coupling. This influences the LHCII excited state lifetime, which is decreased from ~ 2 ns to less than 300 ps (Gray et al. 2022). Most of the studies point to the Chl-a 612, Lutein-620 (Lut-620) pigment pair as the qE site within LHCII (Fig. 2). In fact, a recent theoretical study of different PSII-PSI-LHCII crystals indicates that the Chl-a 612 orientation and distance in relation to adjacent pigments (Chl-a 610, 611) seems to be highly dependent on the protein–protein interactions within the thylakoid membrane that could contain the key to the switch between light-harvesting and quenched states. In that recent study, the *κ*^2^ dipole orientation factor and the distance between pairs of chlorophylls were calculated over many LHCII-LHCI-PSII supercomplexes. (Kim et al. 2022) The short lifetime of Lut-620 S_1_ state (~ 10 picoseconds) can serve as an efficient quencher of the chlorophyll excited state (Duffy et al. 2013). The conformational plasticity of Lut-620 can affect its S_1_ energy level and thus the excitonic coupling with Chl-a 612, in the switch from a light-harvesting to a dissipative state of the LHCII (Artes Vivancos et al. 2020). However, the formation of several other traps for the excess excitation energy cannot be excluded. For example, an orientational change of the neoxanthin carotenoid has been implicated in the switch to the dissipating conformation of LHCII, (Duffy et al. 2014; Liguori et al. 2015) that could take the role of the sensor, rather than a direct quencher (Li et al. 2021).

## Enhanced ΔpH in the LHCII model embedded in a thylakoid membrane

As also reported previously herein, both Zea and PsbS interaction with LHCII might enable protonations of key lumen-exposed residues of LHCII at a pH value of ~ 5.5, which is within the physiological pH range for the lumenal space under photoprotective conditions. We note that LHCII can switch to the dissipating state, given a sufficiently high transthylakoid membrane ΔpH (i.e., lumen pH down to ~ 4.5), (Saccon et al. 2020b) even in the absence of PsbS, or Zea (Rees et al. 1989; Noctor et al. 1991; Walters et al. 1996). There exist sophisticated protocols to identify protonable residues such as constant pH MD simulations, which also allow to account for the interplay between residue pKa and configurational changes. One example is the work of Liguori et al. (Liguori et al. 2019) on pH dependent PsbS conformational changes. However, currently, the application of constant pH MD simulations poses some obstacles for large systems or long timescales and in combination with enhanced sampling techniques (Dobrev et al. 2020). We have employed LHCII trimer models embedded within a native or non-native thylakoid lipid membrane, LHCII-Zea, or LHCII-PsbS models that are very large. In detail, the LHCII, or LHCII-Zea trimer models within a thylakoid membrane patch contain around 137 k atoms (9.8 k for the protein residues, 9.6 k for co-factors, 70.5 k for water molecules and ions, 46.6 k for the all-atom lipids), the LHCII-PsbS models within the thylakoid membrane contain up to around 257 k atoms (17.3 k for the protein residues, 9.6 k for co-factors, 205 k for water and ions, 25 k for the unified-atom lipids). The application of enhanced sampling techniques to reach a qE-relevant timescale in addition complicates the employment of the gold standard technique of constant pH MD for such systems. Thus, the identification of protonable residues at the LHCII lumenal side that are related to qE could be quite tricky computationally and we have chosen not to consider such an approach for the LHCII trimer. In fact, a PsbS-CP29 interaction could increase the pKa value of the minor antenna CP29 residue Glu-128 from 4.46 to 5.24 (Daskalakis 2018). Even the thylakoid membrane thinning we have identified and has been also verified experimentally (Daskalakis et al. 2019b) could contribute to the alteration of the pKa values of the lumen-exposed LHCII residues under photoprotective conditions. Taken together, all these observations render the a priori choice of protonation state of the lumen-exposed residues a difficult puzzle to solve for LHCII modeling at the light-harvesting and dissipating states. Our approach combines the experimental findings (Walters et al. 1996; Liu et al. 2014; Townsend et al. 2018) of possible protonable residues at the lumen-exposed LHCII side, along with the computational propka method (Olsson et al. 2011; Søndergaard et al. 2011) and the effect of a proposed LHC-PsbS interaction on the residue pKa values (Daskalakis 2018). This has led to our choice of protonating the majority of lumen-exposed major LHCII residues (Glu-83, 94, 107, 207 and Asp-111, 211, 215) to simulate the quenched (dissipating) state. We note that whether the LHCII-PsbS, or LHCII-Zea interactions are only affecting the pKa values of LHCII residues, or also influence other qE factors, like the thylakoid membrane fluidity to enable protein–protein interactions, (Daskalakis et al. 2019b) or they allosterically alter the conformation of LHCII is still unclear (Saccon et al. 2020b).

## The LHCII aggregating model—the role of PsbS

In vivo, the molecular basis of qE seems to correlate with a finely tuned interplay between the LHCII intrinsic property to switch between the light-harvesting and the quenched (dissipating) states, its interaction with Zea, PsbS, and the thylakoid lipids (Ruban and Wilson 2020; Azadi-Chegeni et al. 2022). The dynamic nature of the thylakoid membrane with a fine control of protein mobility therein seems also to be correlated with NPQ (Johnson et al. 2011b; Goral et al. 2012; Kirchhoff 2014a, b; Tietz et al. 2020). Photosynthesis relies on this protein mobility as the lateral re-organization of the related proteins (e.g., LHCII), upon sudden changes in illumination, balances the excitation load on PSI, II (Johnson et al. 2011b; Goral et al. 2012; Chukhutsina et al. 2020). PSII-LHCII super-complexes spatially self-arrange into ordered (crystalline) and disordered (fluid-like) domains within the thylakoid membrane in vivo.(Johnson et al. 2011b; Goral et al. 2012) A rigid antenna, anchored within the thylakoid membrane, would severely compromise the switch between efficient light-harvesting and photoprotection, with negative effect on the welfare of the organism (Duffy et al. 2013). Thus, an insight into the atomic scale details of the adaptive flexibility of the membrane is of utmost necessity (Duffy et al. 2013).

The structural re-organization of the thylakoid membrane under photoprotection involves the decrease of protein mobility within an apparent reversible LHCII aggregation (Johnson et al. 2011b). PsbS and Zea promote this LHCII aggregation in vivo (Horton et al. 1996; Johnson et al. 2011b; Goral et al. 2012; Ware et al. 2015; Sacharz et al. 2017). Without being a requirement for the formation of the quenched conformation, (Ilioaia et al. 2008; Malý et al. 2016) the LHCII aggregation could stabilize the dissipative conformation of LHCII, or alter the protein–protein interaction network within the membrane (Krüger et al. 2011a, 2012; Duffy et al. 2013). In fact a change in chlorophyll-xanthophyll interactions has been observed in aggregated LHCIIs, (Barzda et al. 1998) with also a different orientation of the lutein carotenoid between quenched LHCII aggregates and isolated LHCII trimers in the light-harvesting state (Ruban et al. 1997). Aggregation is thus able to change the LHCII scaffold conformation and therefore also the orientation of Chl-a/b and xanthophyll molecules within LHCII (Ruban et al. 1997).

If we assume that a PsbS-LHCII (or Zea-LHCII) interaction is able to significantly increase the pKa values of important LHCII residues at the lumenal side, (Daskalakis 2018) this could possibly be a reason why the qE trigger in vivo lies within lower ΔpH values. In any case, the LHCII-PsbS interaction is crucial, yet no crystal structure exists so far for a well-defined cross-section. It has been proposed that the dimer (inactive) to monomer (active) transition of PsbS and protein–protein interactions within the thylakoid membrane (e.g., CP29-PsbS) are finely tuned by ΔpH and the presence of Zea (Daskalakis 2018; Liguori et al. 2019). We have proposed a model for the LHCII-PsbS complex within a simulated membrane of native thylakoid lipids (Daskalakis 2018; Daskalakis et al. 2019b). The dynamics of the complex were further probed in coarse-grained simulations combined with enhanced sampling methods (Daskalakis et al. 2019b). In the latter study, multiple LHCII copies were embedded within a large scale thylakoid membrane model, and a theoretical description of their mobility was achieved in the presence and absence of PsbS (Daskalakis et al. 2019b). The study was able to reproduce the experimentally observed thylakoid membrane thinning, triggered by ΔpH, (Murakami and Packer 1970a, b; Kirchhoff et al. 2011) which has been correlated with the amplitude of NPQ (Johnson et al. 2011a).

In the previous theoretical description, the thylakoid membrane thinning was observed solely by protonations of residues at the LHCII lumenal side and therefore the thinning might come as a response to the change of LHCII conformation upon protonations (Daskalakis et al. 2019b; Ruban and Wilson 2020). We have to note that LHCII in vivo is partially aggregated (Ruban et al. 1997; Goral et al. 2012). In detail, it was hypothesized that upon enhanced ΔpH, the LHCII protein scaffold changes shape at the lumenal side. In our studies, a thylakoid membrane thinning of ~ 13% has been reported upon transition from neutral to lower lumenal pH, in the presence of PsbS, that can be attributed to changes in the membrane microenvironment (Murakami and Packer 1970a, b; Daskalakis et al. 2019b). The neutral thylakoid lipids DGDG (digalactosyl-diacylglycerol) are accumulated at the lumenal side of LHCII only at low lumenal pH to lower the cost of the induced hydrophobic mismatch between protein-membrane. The latter mismatch could come from either the thylakoid membrane thinning, or from the change of LHCII shape at low lumenal pH. The generation of a locally heterogenous thylakoid membrane (different content of the stromal and lumenal membrane leaflets in the vicinity of LHCII) can be associated with the hydrophobic mismatch, which leads to immobilized LHCII trimers within the membrane, surrounded by the least-diffusive DGDG lipids at the lumenal side (Daskalakis et al. 2019b; Azadi-Chegeni et al. 2022). We have concluded that the LHCII-PsbS complexation enables a re-organization of the thylakoid membrane by increasing the LHCII lateral mobility, by inhibiting the LHCII-DGDG interaction at low lumenal pH. Therefore, the LHCII trimers can aggregate due to solvent (lipid) depletion, or due to the induced hydrophobic mismatch.

In the DGDG-based mobility model, LHCII trimers at low pH seem to exert the lowest value of diffusion coefficient (0.2 × 10^–10^ cm^2^/s), in contrast to the case of neutral lumenal pH with a higher diffusion coefficient (1.9 × 10^–10^ cm^2^/s) (Daskalakis et al. 2019b). PsbS seems to restore the LHCII mobility to higher diffusion coefficient values, even at low lumenal pH. However, in the experimental literature, it is reported that even in the absence of PsbS, the thylakoid membrane re-organization takes place, leading to the full extent of qE, given an enhanced ΔpH (Johnson et al. 2011b; Goral et al. 2012). How can we address this seemingly inconsistent proposals? We must note that at neutral pH the LHCII trimer is surrounded by more monogalactosyl-diacylglycerol (MGDG) lipids (Thallmair et al. 2019). Isolated LHCII are able to force the non-bilayer MGDG lipids to form lamellar phases (Simidjiev et al. 2000). However, at low lumenal pH, because of the altered shape of LHCII identified (Daskalakis et al. 2019b) more DGDG lipids are accumulated only at the lumenal side (Daskalakis et al. 2019b). We note that the MGDG lipids instead can form non-bilayer inverted hexagonal phases within the thylakoid membrane (Dlouhý et al. 2020). At the same time the hydrophobic mismatch is developed between the thylakoid membrane and LHCII. At an enhanced ΔpH and the absence of PsbS, the major LHCII antenna changes its shape by protonations leading to the dissipating state, or the LHCII aggregation due to a pronounced hydrophobic mismatch that develops. The PsbS role in this scenario should then be twofold: (a) to increase the pKa values of lumen-exposed LHCII residues so they can be protonated even at moderate ΔpH values, thus enabling LHCII to change its shape toward the dissipating form, or (b) to increase the mobility of LHCII within the thylakoid membranes, so that LHCII can aggregate which also favors the dissipating form.

Another proposal has recently emerged in the literature for the LHCII-thylakoid lipid interactions (Tietz et al. 2020). The authors of the latter study have suggested that the transition from the light-harvesting (neutral lumenal pH) to the quenched state (low lumenal pH) is associated with an increase of the abundance of the non-bilayer MGDG lipid within the bilayer phase. The latter in turn increases the lateral membrane pressure in the hydrophobic membrane bilayer regions and induces conformational changes in the LHCII trimer that stabilize the dissipating state. This seems to be at odds with the LHCII-DGDG-based mechanism described above, but also with the mechanism of action of the water-soluble enzyme violaxanthin de-epoxidase (VDE) that requires the presence of the non-bilayer lipids and inverted hexagonal phases within the thylakoid membrane, the formation of which is promoted at low pH, for the conversion of Vio to Zea (Latowski et al. 2004; Dlouhý et al. 2020). Possible mechanisms start to unfold that can consolidate these seemingly contradicting findings. The abundance of DGDG lipids around LHCII only at the lumenal side at enhanced ΔpH and in the absence of PsbS, or the presence of MGDG along with the PsbS around LHCII, and even the LHCII aggregation model all come as a response to ease the membrane lateral pressure on LHCII or lower the significant cost of the hydrophobic mismatch, also favoring the dissipating state of LHCII under different conditions. The question remains: what are the associated conformational changes within LHCII, when the dissipating state is favored?

## The LHCII configurational space

So far, several lines of evidence point to ΔpH (and protonations at the LHCII lumenal side) as a factor that should be considered when studying the LHCII conformational transition from the light-harvesting to the dissipating state. In the absence of PsbS, the re-organization of the thylakoid membrane to the dissipating state occurs very slowly; however, higher levels of protonation (enhanced ΔpH) can restore the qE formation (Johnson and Ruban 2011; Saccon et al. 2020b). The light-harvesting and dissipating conformers of LHCII should be equally stable; however, the most thermodynamically favored conformation is the dissipating one at room temperature (Santabarbara et al. 2009). The low pH conformation of LHCII in the crystal structure is highly quenched, (Liu et al. 2004; Pascal et al. 2005; Standfuss et al. 2005). Taken together, we can conclude that the plants cope to keep the LHCII trimer in the light-harvesting conformation for an efficient photosynthetic yield, rather than switching it to the quenched (dissipating) state, which might come naturally as a result of a plethora of stimuli.

Our studies on the LHCII conformational changes have incorporated, since the first time, a thylakoid membrane model under ΔpH and ion gradients (membrane energization), to probe the dynamics of the antenna proteins of PSII at all-atom resolution (Ioannidis et al. 2016; Papadatos et al. 2017; Daskalakis 2018; Daskalakis et al. 2019a, 2020). Although now we can describe the complete configurational space of LHCII, (Daskalakis et al. 2020) under an extensive array of external stimuli (ΔpH, salts, PsbS, carotenoid content), both classical MD and enhanced sampling simulations have linked ΔpH and salt gradients to only minor (subtle) conformational changes within LHCII that could, however, affect excitation and absorption spectra of chlorophyll pigments under photoprotective conditions (Papadatos et al. 2017). Firstly, a conserved sensory domain of the minor CP29 and the major LHCII antenna was identified (helix-D, Fig. 2) that is able to detect chemiosmotic energization, associated with qE conditions (Ioannidis et al. 2016; Papadatos et al. 2017). Residue protonations at the LHCII lumenal side neutralize this helix-D domain, thus enabling its position closer to the LHCII hydrophobic core, and away from the lumenal aquatic phase; a conformational change that seems to be enhanced in the presence of Zea under photoprotective conditions (Papadatos et al. 2017). The movement of helix-D has been proposed to induce quenching in the conformation of the crystal structure of LHCII. (Yan et al. 2007) Allostericity on a network of LHCII residues (Thr-57, Asn-61, Glu-65, Arg-185, Phe-189, Gly-193, Gln-197, and Thr-201), has also been identified within the LHCII trimer, and can play a major role in the switch between light-harvesting and dissipating states (Daskalakis et al. 2019a; Li et al. 2020). On this consensus, another response of LHCII to membrane energization comes as a change of the helix A/B interhelical crossing angle (Daskalakis et al. 2019a, 2020; Li et al. 2020). By employing a comprehensive array of external stimuli (ΔpH, PsbS, Zeaxanthin, salt gradients) the complete configurational space of LHCII was revealed (Daskalakis et al. 2020). The latter includes conformational transitions in the following LHCII domains: helix-D, the C-terminal, the N-terminal, and the helix A/B orientations (Fig. 2), in line with previous suggestions in the literature (Yan et al. 2007; Liguori et al. 2015; Papadatos et al. 2017; Daskalakis et al. 2019a; Cupellini et al. 2020; Li et al. 2020). A recent study on the CP29 minor antenna (Cignoni et al. 2021) confirms that helix A/B and helix-D subtle conformational changes are transitions that are evident in the LHC protein scaffold by employing enhanced sampling molecular dynamics techniques. In detail, α scissoring motion of helices A and B of the major LHCII, along with the movement of helix-D (Fig. 2) toward the hydrophobic inner side of the protein scaffold can induce an adequate conformational change and bring Chl-a 612 and Lut-620 closer together (Daskalakis et al. 2019a, 2020; Li et al. 2020). The dissipation of energy within the antenna follows due to the increased excitonic coupling in the Chl-a 612/Lut-620 pigment pair (Daskalakis et al. 2019a, 2020; Maity et al. 2019; Li et al. 2020). However, this motion and effect on the latter pigment pair is not sufficient to describe the full extent of qE (Daskalakis et al. 2019a, 2020; Gray et al. 2022). A more elaborate description of the short-range interactions within the pigment network of LHCs might be necessary (Cignoni et al. 2021; Gray et al. 2022). The motion of helix-D toward the inner hydrophobic core of LHCII could also be responsible for the change in the shape of LHCII at the lumenal side, triggering the accumulation of the DGDG lipids therein. In a recent study (Li et al. 2020) the authors have suggested that the fluorescence quenching is triggered by the change of the Glu-94 hydrogen bond partner from Lys-99 to Gln-103, due to the lumen acidification and the Glu-94 protonation (Fig. 2), that favors the dissipating conformation of the major LHCII trimer. This conformational change also involves the reduction of the average distance between helices E and D of the major LHCII trimer (Fig. 2), along with the scissoring motion of the A/B helices. The change in the helix E/D distance might also correlate with the change in the shape of LHCII at the lumenal side and the accumulation of the DGDG lipids described above.

**Fig. 3:**
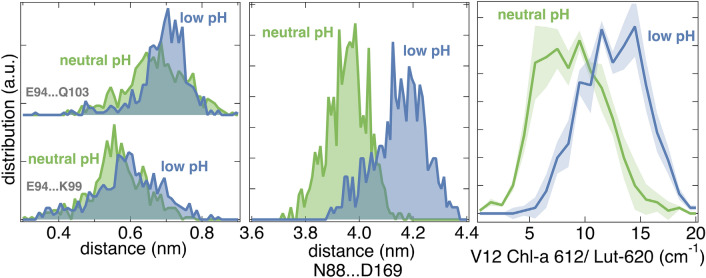
**A** Histograms of the distances between Glu-94 (E94) to either Lys-99 (K99), or Gln-103 (Q103) at neutral (green) and low (blue) lumenal pH. **B** Histogram of the distances between Asn-88 (N88) and Asp-169 (D169) as an indicator of the helix-A/B conformational change in LHCII at neutral (green) and low (blue) lumenal pH. **C** The excitonic coupling values for the Chl-a 612, Lutein-620 pigment pair in cm^−1^ at the associated neutral (green) and low (blue) lumenal pH cases of **A** and **B** as averages over the three monomers per trimer. The standard deviation in the histogram (calculated out of the three monomers in the trimer) is shown as shade for the respective pH states

**Fig. 4:**
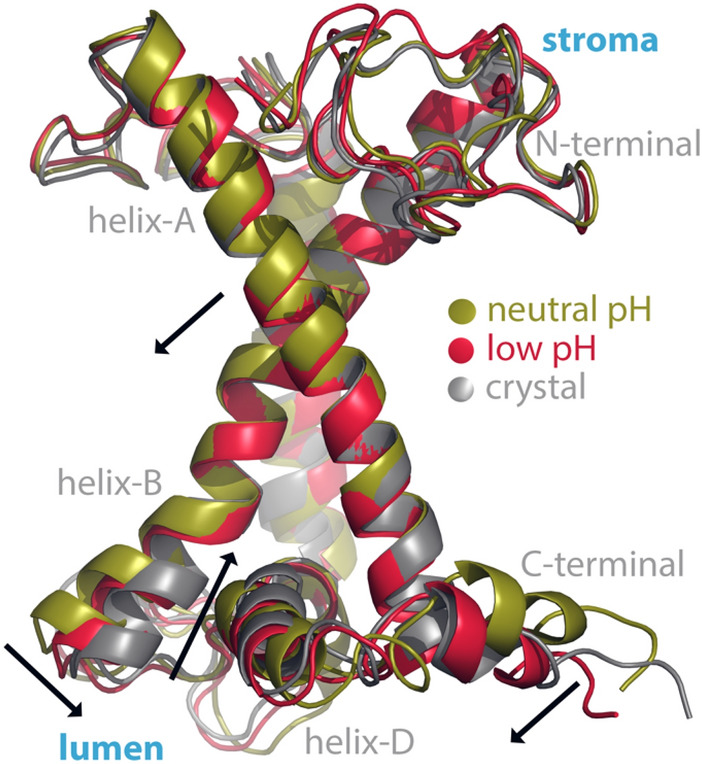
Three conformations of a monomer of the LHCII trimer at neutral pH (olive-green), low lumenal pH (red), and at the crystal structure (gray, chain C, pdb ref 1RWT). Selected domains are shown, along with black arrows at the lumenal side that indicate a proposed conformational transition of the major LHCII from the light-harvesting to the dissipating state

We have extended up to 3 μs each, the equilibrium trajectories described in ref (Daskalakis et al. 2020) for the major LHCII trimer at neutral and low lumenal pH, based on the amber force field and the ad hoc description of the carotenoid dynamics (Duan et al. 2003; Prandi et al. 2016). One replica per model (neutral, low lumenal pH) has been run up to 3 μs. The LHCII trimer has been embedded in a membrane patch of native thylakoid lipids described by the Amber force field (Wang et al. 2004; Retegan and Pantazis 2017; Daskalakis et al. 2020). The computational details are identical as in ref (Daskalakis et al. 2020). The extended trajectories were analyzed (Figs. 3A–C) in terms of the distribution of distances between Glu-94 (CD atom) and either Lys-99 (NZ atom), or Gln-103 (NE2 atom), as shown in Fig. 3A. The results show a broad distribution of distances in both pH states and transient formation of hydrogen bonding between Glu-94 (E-94) and Lys-99 (K-99), or Gln-103 (Q-103) in line with the study of (Li et al. 2020). Instead, the histograms of the distances between Asn-88 (N-88, Ca atom) and Asp-169 (D-169, Ca atom) along the same equilibrium trajectories are shown in Fig. 3B, as a clear indicator of the helix A/B conformational change described above. These residues were chosen because Asn-88 faces the lumenal side (helix-B), whereas Asp-169 (helix-A) faces the stromal side of LHCII (Fig. 2). A striking difference emerges, as at low pH there is a rather distinct shift of the distribution to higher distances between Asn-88 and Asp-169 (Fig. 3B). In Fig. 4 two structures of a LHCII monomer are shown and correspond to the light-harvesting state at neutral pH (olive-green), the dissipating state at low pH (red) as the middle structures of the most populous clusters out of the equilibrium trajectories extracted by employing the Patrick-Jarvis method in GROMACS (Berendsen et al. 1995). The crystal structure (chain C, pdb ref 1RWT) (Liu et al. 2004) is also shown in gray for reference (Fig. 4). Remarkably, the LHCII conformation at low pH matches well with that of the crystal structure, in line with the literature (Liu et al. 2004; Pascal et al. 2005; Standfuss et al. 2005). Arrows indicate the proposed transition from the light-harvesting state to the dissipating one. This includes a motion of helix-D toward the inner hydrophobic core of the LHCII trimer and an orientational change of helices A and B, shown mainly enhanced for the lumenal side (Daskalakis et al. 2019a, 2020; Li et al. 2020).We provide the structures in Fig. 4, where the Asn-88 and Asp-169 distance is at 4.087 nm (crystal structure), at 4.072 nm (low pH simulated state), and at 3.924 (neutral pH simulated state). Please note that the clustering algorithm employed for the LHCII trajectories, the calculation of the most populous clusters and the associated average structures (Fig. 4) relied on configurational criteria for the protein helices and not only on the Asn-88 to Asp-169 distance. Therefore, while we observe deviations between the distances reported in Fig. 3B and those for Fig. 4, there is a clear trend. The distributions of the distances between Asn-88 and Asp-169 (Fig. 3B) certainly justify the proposal of an induced hydrophobic mismatch in the thylakoid membrane at enhanced ΔpH, in combination also with helix-D motion (Fig. 4).

We have calculated the excitonic coupling between Chl-a 612 and Lut-620 within the LHCII trimer along the aforementioned 3 μs extended long equilibrium trajectories at neutral and low lumenal pH, based on the TrESP method, (Madjet et al. 2006) as described in detail elsewhere (Daskalakis et al. 2019a; Maity et al. 2019). The results are depicted in Fig. 3C, where the excitonic coupling between Chl-a 612 and Lut-620 appears to be below 10 cm^−1^ at the neutral pH case, and above 10 cm^−1^ at the low lumenal pH case (Fig. 3C), indicating a trend in couplings consistent with what we would expect for a quenching site within LHCII (Balevičius et al. 2017). We have to note that the rate constant for an excitation transfer from a donor (Chl-a 612) to an acceptor (Lut-620) is proportional to the square of the excitonic coupling between them. Of course, the small coupling differences cannot be associated with a hard switch that would have certainly elaborated on the full extent of the qE mechanism, in line with refs (Cignoni et al. 2021; Gray et al. 2022).

## Rigidity of the LHCII protein scaffold might allow only subtle changes in the pigment network

In our most recent computational studies, (Daskalakis et al. 2019a, 2020) we have so far failed to identify a hard conformational switch between Chl-a 612/Lut-620 excitonic coupling values that would justify the transition of LHCII from the light-harvesting to the dissipating state, in line with ref (Cignoni et al. 2021). This might be due to either the inadequate level of theory employed so far for the description of the inter-pigment interactions that involve carotenoids, (Maity et al. 2019; Cignoni et al. 2021; Gray et al. 2022) or because we have not so far considered an extended response from the whole pigment network within LHCII, given the conformational changes identified (Daskalakis et al. 2020). Another explanation could be the possible CT character of the Chl-a/Lut-620 interaction under photoprotective conditions, (Cupellini et al. 2020) or the formation of CT states between chlorophylls, or chlorophyll-zeaxanthin pigments, (Ostroumov et al. 2020) that have to be addressed in future studies, within the complete configurational space proposed for the LHCII trimer (Daskalakis et al. 2020). Rigidity in the LHCII protein scaffold might be due to the increased ratio of chlorophylls to carotenoids embedded within the complex. This rigidity enables only subtle changes in the pigment network within LHCII. To the contrary of what has been so far simulated for the LHCII of higher plants (i.e., our research on LHCII from spinach and pea), we have reported a rather flexible protein scaffold for another light-harvesting antenna complex, that of the diatoms, the Fucoxanthin and Chlorophyll-a/c binding Protein (FCP)—with an increased carotenoids to chlorophylls ratio (Chrysafoudi et al. 2021). Diatoms are found in fresh water and oceans, they are unicellular eukaryotic microalgae in the red lineage of photosynthetic organisms with remarkable light-harvesting capabilities, but also with a very efficient photoprotective mechanism for their adaptation to the fluctuating light at the ocean surfaces (Lepetit et al. 2017; Wang et al. 2019). The interaction of FCP, with the photoprotective LHCX1 family of proteins in diatoms, the latter being the analog of PsbS of higher plants, (Giovagnetti and Ruban 2018) leads to a nine-fold decrease of the chlorophyll excited state lifetime in FCP. The Fucoxanthin-301/Chlorophyll-a 409 pigment pair has been proposed to be responsible for such a remarkable switch of the FCP between the light-harvesting and the quenched (or dissipating) states. This latter raises the question whether the photoprotective mechanism in higher plants is a multi-component and rather complex response; a response that includes protein–protein interactions, thylakoid lipids, and the inter-pigment network in a rather rigid LHCII with a higher content of chlorophylls. These possibly cannot be all probed at the same time by the computational models that consider only a few parameters in a finite system-size and time limit. On the other hand, the flexible FCP scaffold in diatoms with fewer chlorophylls participates in a less complicated response that can be fully probed by simpler computational models and at shorter related timescales.

## Conclusion and future perspective

**Fig. 5:**
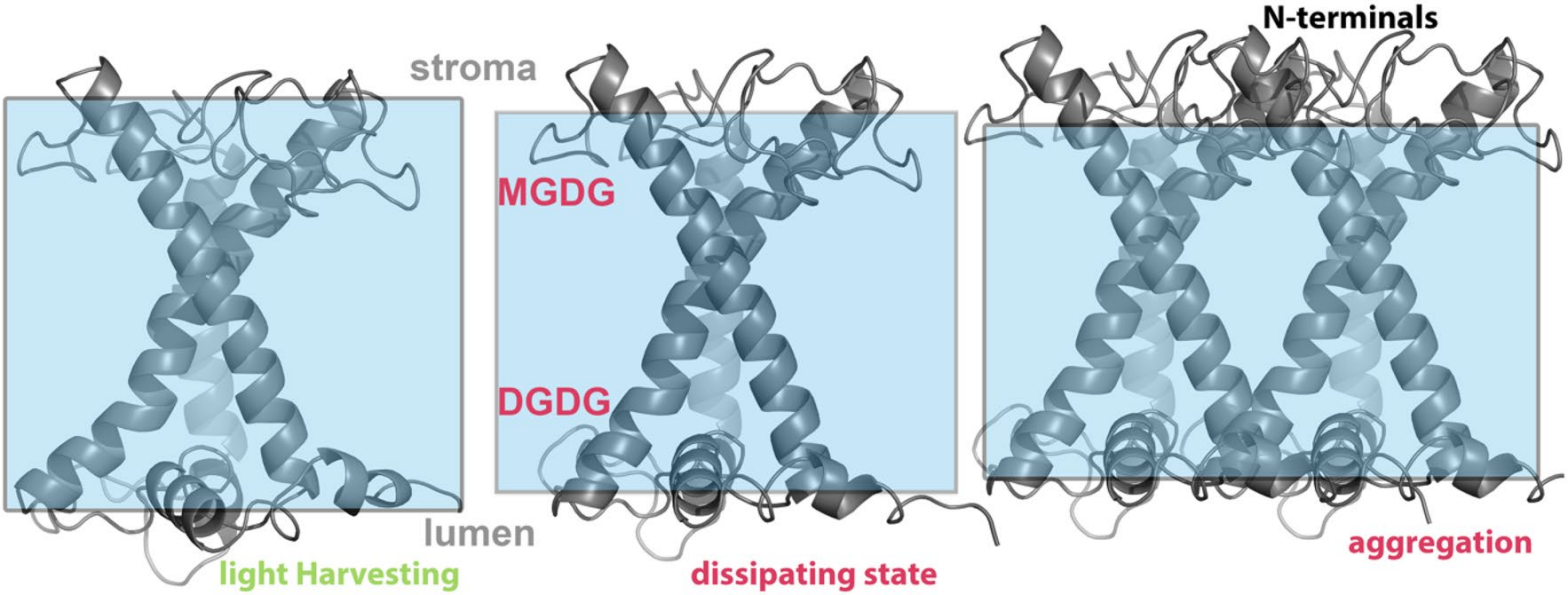
Conformational changes in the LHCII scaffold (gray cartoons) upon the transition from the light-harvesting to the dissipating configuration. The thylakoid membrane is depicted as light blue rectangles, whose width (y-axis) roughly indicates the relative membrane thickness. In the dissipating state (middle) more DGDG lipids are accumulated at the lumenal space, whereas more MGDG lipids are found in the stromal space and the vicinity of LHCII scaffold. In the aggregation state, aggregated LHCII monomers instead of the actual LHCII trimers are shown for illustrative simplification

The major LHCII antenna of higher plants has an intrinsic property to switch between light-harvesting and dissipating states (conformations) to protect the photosynthetic apparatus from oxidative stress. This intrinsic switching function is primarily related to protonations of certain lumen-exposed residues. These protonations are possible when the pH value at the lumen drops to around ~ 5.5 associated with a transthylakoid proton gradient (ΔpH≈1.0–1.5). Within the crowded environment of the thylakoid membrane the latter protonations are favored within the physiological pH of 5.5 in the presence of Zea (the xanthophyl cycle) and the photoprotective PsbS protein. Without the latter two the protonations would require a very low pH (~ 4.5) at the lumen space, which would denature the thylakoid membrane. We cannot exclude the possibility that a PsbS-LHCII interaction not only enables protonations at lumen-exposed residues, but also conformational changes within LHCII that favor the dissipating state. We have introduced such effects in our models first indirectly by a priori protonating the majority of LHCII residues at the lumen side for the isolated LHCII complexes embed in a model thylakoid membrane, and directly by building LHCII-PsbS/LHCII-Zea models. Under photoprotection, LHCII switches to a conformation by a helix-D motion toward the inner LHCII structure, and by an increase in the distance between residues at the helix A/B stromal (D169) and lumenal (N88) sides, respectively. The latter increase in the D169-N88 distance was identified based on the new simulations and analysis reported in this manuscript; however, the associated scissoring-like motion of helices A/B has been proposed previously from our group (Daskalakis et al. 2019a). The conformation of LHCII under photoprotection triggers the accumulation of more DGDG thylakoid lipids at the lumenal face of the membrane around LHCII at enhanced ΔpH. The accumulation of specific thylakoid lipids around LHCII could certainly affect the thylakoid membrane properties, like the overall thickness. MDGD and DGDG lipids have different lengths. So, the enrichment of the thylakoid membrane in MGDG lipids, as more DGDG lipids are bound to the trimer at the luminal space, could also explain the overall membrane thinning observed. An average thylakoid membrane thinning with locally heterogeneous upper–lower membrane leaflets in the vicinity of LHCII (Fig. 5) comes thus to counteract the cost of the developed hydrophobic mismatch. The membrane thinning could also lead to exposure of the LHCII stromal sides that leads to aggregation involving interactions between their N-terminals enabling an efficient protein–protein association (Fig. 5). Finally, we must pinpoint that modeling might be unable to fully reproduce the dynamics of LHCII complexes within the crowded environment of a thylakoid membrane. Protein–protein associations or PSII-LHCII super-complex formation could become also crucial in stabilizing the light-harvesting or the quenched (dissipating) conformation. One would also expect significant differences between the simulated activation energies and the in vivo case associated with the transition between different LHCII conformations, even with a comprehensive array of external stimuli for the LHCII configurational space (Daskalakis et al. 2020). The a priori protonation state of the LHCII employed in our models might have also introduced artifacts in the simulations. Nevertheless, we expect that modeling enables at least a partial insight at all-atom resolution into the gray or obscure areas defined in the experimental literature. In any case, we might face a situation where numerous photoprotective mechanisms have been developed throughout the LHCII evolution, with different pieces as main or side mechanisms to be revealed by molecular simulations, or the experimental literature, dependent on the conditions, while the greater puzzle picture remains to be resolved (Papadatos et al. 2017). Large scale simulations of the whole photosynthetic membrane of higher plants, and especially the protein–protein interactions therein could enable a future accurate insight into the onset (qE) and development of the NPQ mechanism in higher plants.
